# Innovative strategies in genitourinary cancer: the role of oncolytic viruses

**DOI:** 10.3389/fonc.2024.1461324

**Published:** 2024-10-11

**Authors:** Jie Zhang, Kepu Liu, Zheng Zhu, Shihao Shang, Di Wei, Yu Zheng, Lei Zhang, Ying Liang, Dongen Ju, Jianlin Yuan

**Affiliations:** ^1^ College of Life Sciences, Northwest University, Xi’an, Shaanxi, China; ^2^ Department of Urology, Xijing Hospital, Fourth Military Medical University, Xi’an, Shaanxi, China; ^3^ Precision Pharmacy & Drug Development Center, Department of Pharmacy, Tangdu Hospital, Fourth Military Medical University, Xi’an, Shaanxi, China

**Keywords:** oncolytic virus, renal cancer, bladder cancer, prostate cancer, tumor, therapy

## Abstract

Urinary tumors pose a significant health threat because of their high prevalence and recurrence rates. Despite the availability of various treatment options, many patients poorly respond to traditional therapies, highlighting the urgent need for alternative approaches. Oncolytic viruses are promising therapeutic agents. These viruses exploit the unique characteristics of cancer cells to specifically target and destroy them, thereby triggering potent antitumor immune responses. This review delves into recent advancements and future prospects of oncolytic viruses, focusing on their application in renal, bladder, and prostate cancers. By discussing practical implications and the potential of different viruses, including the cowpox virus, adenovirus, measles virus, coxsackievirus, and reovirus, we pave the way for further exploration and refinement of this exciting field.

## 1 Introduction

As a serious threat to human health, urological tumors require diverse treatment methods. Traditional treatment approaches, including surgery, chemotherapy, and radiotherapy, control the disease to some extent (1). Onset may be associated with smoking, obesity, insulin resistance, hypertension, and chronic kidney disease. However, issues, such as high recurrence rates and significant side effects, continue to affect the medical community and patients. For instance, renal cell carcinoma (RCC), a malignant tumor originating in the kidneys, is generally treated by surgical removal (2). However, for patients with metastatic RCC, surgery often has a limited efficacy, making adjuvant and targeted therapy crucial supplements (3). Renal cell carcinoma encompasses a range of histopathological entities, with the most common subtypes being clear cell renal cell carcinoma (80%), peroid renal cell carcinoma (13-20%), and chromophilic renal cell carcinoma (5%) (4). For localized renal cell carcinoma that has not metastasized, the standard treatment is surgical resection, and radiofrequency ablation, cryoablation, and stereotactic ablative radiotherapy may be used when the patient has a high surgical risk, is weak, has isolated kidneys, has impaired baseline renal function, or has multiple bilateral tumors (5). Bladder cancer is the 10th most common malignancy worldwide (6). Onset is often related to factors such as smoking, air or water pollution, dietary patterns and medical conditions (7). Based on the depth of invasion, BC is divided into non-muscle-invasive bladder cancer (NMIBC) and invasive bladder cancer (MIBC). Transurethral resection of bladder tumors (TURBT) is the standard of care for NMIBC. For non-muscle-invasive bladder cancer (NMIBC), intravesical therapy (primarily BCG) plus maintenance therapy is the mainstay of treatment to prevent recurrence and progression after initial TURBT; For those patients who do not respond to BCG, additional treatment is required. For localized MIBC, optimizing care and reducing morbidity after cystectomy are important goals (8). Urothelial carcinoma (UC) is the major subtype of bladder cancer, and the first-line treatment for patients with locally advanced urothelial carcinoma is cisplatin-based chemotherapy (9). Prostate cancer affects millions of men worldwide, mainly in areas with high human development indices (10). The main contributing factors for prostate cancer include genetics, obesity, physical activity, and smoking (11). The treatment of prostate cancer (PC) is complex. Localized PC can be managed with active surveillance, radiotherapy, or prostatectomy. Androgen deprivation therapy (ADT), salvage radiotherapy, and chemotherapy are the primary treatment methods for recurrent or metastatic PC (12). Although these traditional approaches increase the patient survival and improve the quality of life, they have limitations. Surgical trauma, the toxic side effects of chemotherapy, and damage to normal tissues due to radiotherapy cannot be ignored. More importantly, these methods have relatively high recurrence rates, especially in the case of advanced or metastatic tumors, and treatment outcomes are often unsatisfactory (13).

In recent years, the rapid development of biotechnology has brought new hope to the treatment of urological tumors in the form of oncolytic viruses (OVs), representing an emerging therapeutic strategy (14, 15). OVs selectively infect and kill tumor cells by stimulating the body’s own antitumor immune response to achieve therapeutic goals (16). Compared with traditional treatment methods, OVs offer a higher targeting specificity and fewer toxic side effects. More importantly, OVs activate the patient’s immune system, forming long-term immune memory, thereby effectively preventing tumor recurrence and metastasis (16).

OV research can be traced back to the late 19th century when doctors observed cases of tumor regression coinciding with viral infections (17). However, because of scientific and technological constraints, this discovery could not be promptly converted into an efficacious treatment modality (17). With the rapid development of modern biotechnology, our understanding of OVs has deepened and their application in cancer treatment has gradually moved from theory to practice. It has been shown that OVs have tremendous potential for the treatment of urological tumors. Researchers have developed various targeted OV strains for different types of urological tumors including RCC (18), BC (19), and PC (20). These viral strains can replicate efficiently within tumor cells and cause cell lysis as well as activate the body’s immune system by releasing tumor-associated antigens, forming a powerful antitumor immune response.

Overall, OVs provide new ideas and methods for the treatment of urological tumors. Although research in this field is in its early stages, excellent results of preclinical studies and preliminary clinical trials bring hope to patients with urological tumors (13). We believe that OVs will play an important role in the future treatment of urological tumors.

## 2 Overview of oncolytic viruses

**Figure 1:**
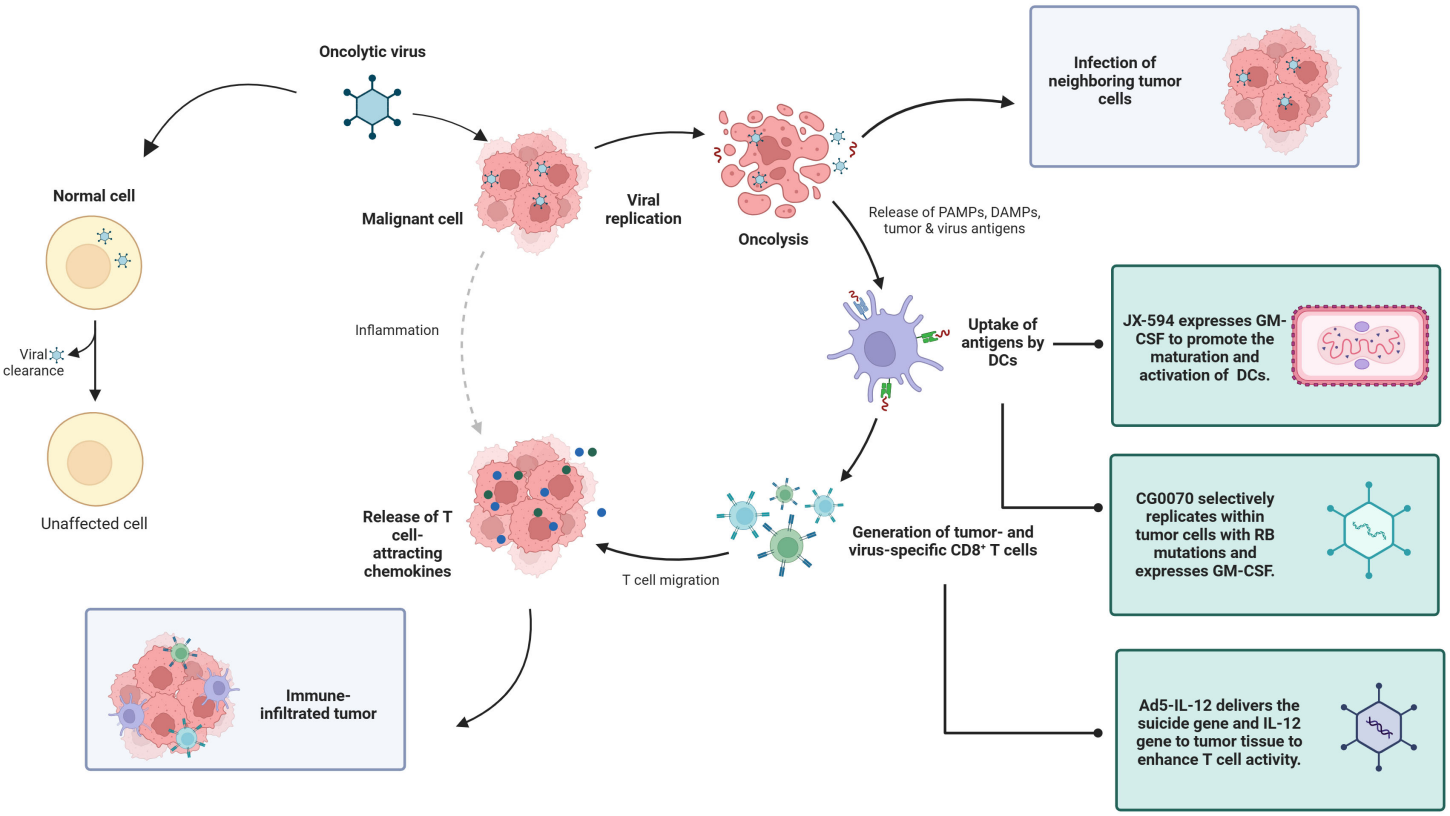
Properties of oncolytic viruses.

OVs, as a new type of biological therapy, selectively replicate and lyse within tumor cells, causing immunogenic cell death and subsequently inducing antitumor immune responses. It is generally anti-tumor in four ways: including oncolysis, anti-tumor immunity, transgene expression, and vascular collapse (21). First, the replication of the virus in cancer cells can induce cell lysis, and the viral replication leads to a continuous increase in the viral dose, which is more lethal to the tumor, and at the same time, proteins are also produced during the viral replication process, which are also toxic to tumor cells (22). The third mechanism by which oncolytic viruses mediate tumor cell destruction is through the induction of non-specific and specific anti-tumor immunity (23). Finally, oncolytic viruses can greatly increase the sensitivity of tumor cells to chemotherapy and radiation therapy (24). Because of these advantages, we chose OVs for discussion. OV therapy has advantages, such as strong targeting, relatively few side effects, and the ability to improve the efficacy through the genetic engineering of viruses, providing new ideas for the treatment of genitourinary tumors (25). A review of 97 published OV trials reported that most OVs tested have used large deoxyribonucleic acid (DNA) viruses such as adenovirus, HSV-1, reovirus, and poxviruses (26) (**Figure 1**).

### 2.1 Vaccinia virus

VACV is an enveloped double-stranded DNA orthopoxvirus that only replicates in the cytoplasm. VACV has a large genome (~190 kb) that stably expresses at least 25 kb of exogenous therapeutic genes in a single vector (**Table 1**). Since the late 1980s, recombinant DNA technology has been used to explore the utility of recombinant VACV and other poxviruses as vectors for active immunization in cancer and infectious disease settings (27). VACV has natural tumor tropism and potential for systemic administration. It has a rapid replication and lysis cycle. The virus is released from infected cells within 8 h of infection and destroys infected cells within 48–72 h post-infection (28). VACV has been used as a smallpox vaccine for many years, and adverse effects have occurred less frequently (29). In terms of being used as an oncolytic virus, firstly, VACV has a certain safety, which is mainly manifested in the fact that it only replicates in the cytoplasm and does not participate in the host’s genes; Second, VACVs have a natural tumor tropism, which means that they are able to localize naturally to tumor tissues and have the potential to do so through systemic administration. At the same time, the replication cycle of VACVs is fast and lytic, which allows them to proliferate rapidly and release rapidly after infecting host cells; In addition, a notable feature is their ability to replicate under hypoxic conditions, increasing their adaptability in the tumor microenvironment; Finally, because VACVs have no receptor restrictions on their entry into host cells, they exhibit high infectivity not only in various host species, but also in a wide range of tissue types, which facilitates their use in a variety of preclinical studies (30). At present, VV has been intensively studied in many preclinical and clinical studies, such as the NOV virus applied in colorectal cancer, which increases antitumor activity by replacing the vTk and VGF regions with TRAIL and Ang1 (31).

**Table 1:** Properties of oncolytic viruses.

Virus Type	Attribute
Smallpox virus	Enveloped double-stranded DNA virus
Enterovirus	Positive-sense single-stranded RNA virus
Measles virus	Enveloped single-stranded negative-sense RNA virus
Norovirus	Non-enveloped double-stranded RNA virus
Adenovirus	Non-enveloped double-stranded linear DNA virus

### 2.2 Encephalomyocarditis virus

The encephalomyocarditis virus (EMCV) is a single-stranded RNA picornavirus with a broad host range that infects various mammals and birds (32) (**Table 1**). EMCV causes sudden death, myocarditis, encephalitis, neurological disorders, and diabetes. However, when EMCV infects people, it causes only mild disease (33). EMCV also can inhibit apoptosis and induce inflammatory reactions, which may play a role in tumor suppression (33). Unlike many viruses, which may not be able to overcome hypoxia-mediated inhibition of protein synthesis for viral replication, hypoxia or increased HIF activity common in solid tumors may inadvertently exacerbate EMCV replication and virulence due to various oncogenic mutations on general oxygen-sensitive pathways or a growing list of genes such as PTEN, TSC, and VHL. Although EMCV is currently primarily used in the treatment of kidney cancer, its potential as an oncolytic virus may extend far beyond kidney cancer, suggesting that it could have therapeutic potential for a variety of tumor types (34).

### 2.3 Measles virus

MV is an enveloped single-stranded negative-sense RNA virus belonging to the genus Morbillivirus of the family Paramyxoviridae (13) (**Table 1**). The anticancer properties of MV were first discovered in 1949 when wild-type MV infection led to the regression of Hodgkin’s lymphoma. Oncolytic MV is an attenuated vaccine strain derived from the Edmonston-B (MV-Edm) vaccine lineage, which has been demonstrated to be safe and efficacious for cancer treatment in preclinical *in vitro* and *in vivo* studies (35). The MV receptor nectin-4 is abundantly expressed in lung, colon, ovarian, and breast cancers, making it a potential tumor marker (36). In addition to the tropism of MV to specific cell receptors, other underlying mechanisms contribute to the tumor selectivity of MV vaccine strains, such as defects in the IFN antiviral response pathway, which is often dysregulated in tumor cells to facilitate their escape from the host immune system (37). There are currently many open and recruiting MV clinical trials: such as Modified Measles Virus (NCT02962167) for Recurrent Medulloblastoma or Recurrent ATRT, Measles Vaccine (NCT00828022) for Non-Small Cell Lung Cancer, and Progressive, Recurrent, or Refractory Ovarian Epithelial Cancer or Primary Peritoneal Cancer’s Recombinant Measles Virus Vaccine (NCT00408590) and many more. Despite some clinical trials of oncolytic measles viruses, there is only one clinical trial underway involving an oncolytic measles virus expressing pro-inflammatory transgenes (38).

### 2.4 Reovirus

Reovirus is a non-enveloped double-stranded RNA virus that was initially isolated from the respiratory or intestinal tract of humans and animals (**Table 1**). However, it is not associated with any disease (except for infection in rodents and birds, generally not causing notable disease, especially in adult animals). The Reoviridae family consists of six genera among which orthoreoviruses can infect both animals and humans (14, 15).

Ras-activated tumor cells are effectively killed by reovirus, possibly due to double-stranded RNA-dependent kinase (PKR) inactivation, and efficient translation of viral proteins occurs in Ras-activated tumor cells, allowing for efficient production of progeny viruses. Reovirus was originally thought to function primarily through apoptosis (39). Apoptotic signals commonly exhibited by infected cells include IFN production and NF-κB activation, cytoplasmic dsRNA detection by PKR, retinoic acid-inducible gene I (RIG-I), or melanoma differentiation-associated protein 5 (MDA5), or inflammatory cytokines (e.g., TNF-associated apoptosis-induced ligand) in response to NF-κB and/or IRF3 signaling after σ1 and μ1 receptor binding or membrane penetration, TRAIL, which binds to surface death receptors and triggers activation of caspase-3 and -7. Blocking apoptotic caspases does not always eliminate reovirus-induced cell death, and necroptosis depends on viral dsRNA recognition and induction of type I IFN responses, as well as autophagy following acute endoplasmic reticulum (ER) stress, which have been identified as alternative modes of reovirus-induced cell death (40). Reovirus T3D is the most widely used oncolytic virus therapy (OVT). It is currently available for the treatment of glioma, ovarian, pancreatic, peritoneal, and gastric cancers, with the initial trial using reovirus as monotherapy, mostly given intravenously; There were almost no serious adverse events, and safety was demonstrated (41). Reovirus, as an OV therapeutic, has shown certain efficacy in preclinical models, but has been effective in only a minority of patients in clinical applications (40).

### 2.5 Adenovirus

Ad belongs to the non-enveloped virus class and contains a linear double-stranded DNA genome with a diameter of ~950 Å within a twenty-sided icosahedral capsid (19) (**Table 1**). Adenoviruses are relatively easy to produce in high titer and high purity, making them one of the most commonly used viral vectors for applications ranging from gene and cancer therapy to vaccine development (20). The E1A gene is the first gene to be expressed at the time of viral infection and is essential for the expression of all subsequent viral genes. Therefore, E1A deletion is commonly used to generate replication-deficient adenoviral vectors. In contrast, CRA is produced by mutating the E1A gene or replacing the native E1A promoter with a cancer-specific promoter to alter E1A expression. Because adenovirions can pack up to 105% of the length of the wild-type genome, it is common to remove certain parts of the viral gene that are necessary for virion formation, such as the E3 region, to insert the therapeutic gene into the recombinant adenovirus genome (42). At present, there are many kinds of adenoviruses that have entered clinical trials, including but not limited to bladder cancer, prostate cancer, and kidney cancer, and CG0070 has been administered intravesically to treat bladder cancer with good therapeutic results (43).

## 3 Application of oncolytic viruses in different genitourinary tumor treatments

### 3.1 Application in renal cell carcinoma treatment

**Figure 2:**
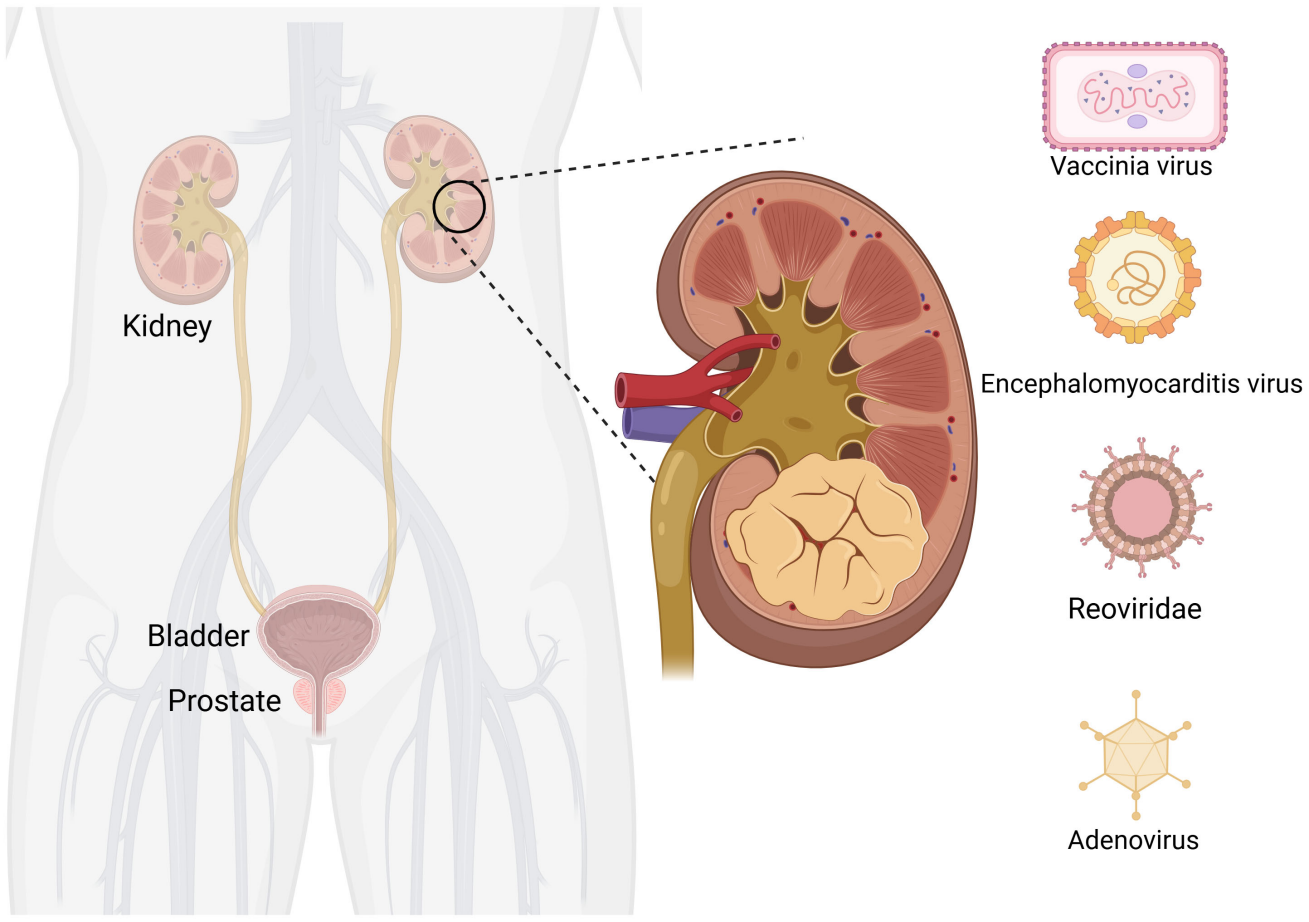
Oncolytic viruses are common in kidney cancer. Created with Biorender.com.

RCC accounts for 3% to 5% of adult malignancies. The incidence is increasing annually in most countries, but the mortality rate is decreasing in developed countries (44). The etiology of RCC remains unclear, but it is associated with genetics, smoking, alcohol consumption, obesity, hypertension, antihypertensive drugs, and diabetes (45). Currently, treatment options for RCC mainly include targeted therapy drugs such as sorafenib, sunitinib, and pazopanib; immunotherapy drugs in combination therapy; and immune checkpoint inhibitors in combination with targeted drugs; or immune checkpoint inhibitors (46). Although drug therapy has shown good efficacy, cases of patient intolerance, significant side effects, or moderate treatment effects are known. Therefore, the development of new antitumor drugs is necessary and OVs have a great development potential as emerging treatment modality (**Figure 2**).

#### 3.1.1 Vaccinia virus and renal cell carcinoma

VACV is considered to be an OV for RCC. It is generally used in combination with tumor drugs to enhance the efficacy or is modified to better target cancer cells. JX-594 is a thymidine kinase (TK) gene-inactivated oncolytic vaccinia virus expressing granulocyte-macrophage colony-stimulating factor (GM-CSF) and lac-Z transgenes, and is the most widely used oncolytic vaccinia virus in clinical trials (47),designed to destroy cancer cells by replication-dependent cell lysis and stimulation of anti-tumor immunity (48). For example, Park et al. combined the oncolytic VACV JX-594 with programmed cell death protein-1 (PD-1) inhibitors to reshape the cellular environment into a tumor-suppressive environment, effectively reducing the primary tumor and metastatic burden and reducing liver damage (49). Similarly, they evaluated the efficacy of systemic JX-594 monotherapy versus sunitinib monotherapy in a mouse model of metastatic orthotopic RCC as early as 2022. Compared with sunitinib monotherapy, systemic JX-594 monotherapy yielded significantly better treatment outcomes when cold tumors were converted to hot tumors, demonstrating better therapeutic efficacy in early and late mRCCs. Sunitinib monotherapy effectively inhibited early mRCC primary tumor growth and lung metastasis (50).

To increase the toxicity of the VACV to renal tumor cells, Fend et al. constructed a novel strain of VACV and verified its ability to inhibit tumor growth in models (64). This virus was constructed by deleting the thymidine kinase (TK) and ribonucleotide reductase (RR) genes and expressing the fusion suicide gene FCU1, which is derived from yeast cytosine deaminase and uracil phosphoribosyltransferase genes. In a xenograft mouse model, the percentage of tumor tissue necrosis in mice injected with this virus was higher than that in the control group. Currently, three oncolytic viruses derived from the cowpox virus have entered clinical application: Pexa-VEC combined with Cemiplimab is used in Phase I/II clinical trials, while JX-594 and TBio-6517 are used individually in Phase I clinical trials (**Table 2**).

**Table 2:** Clinical trials of oncolytic viruses.

Virus species	Virus Name	Type of cancer	Conbination	Mode of administration	Phase	NCT
VV	Pexa-VEC	RCC	Cemiplimab	Intravenous administration	I/II	NCT03294083
VV	JX-594	solid tumors	Null	Intravenous administration	I	NCT00625456
VV	TBio-6517	solid tumors	pembrolizumab	Subcutaneously injection	I	NCT02432963
VV	TBio-6517	solid tumors	Pembrolizumab	Intratumoral injection	1/2a	NCT04301011
VV	VET3-TGI	solid tumors	pembrolizumab	Intratumoral injection or Intravenous administration	I	NCT06444815
VV	PF-07263689	solid tumors	sasanlimab	Intravenous administration	I	NCT05061537
Ad	adenovirus-transfected DC	RCC	CIK	Not applicable	I/II	NCT01924156
Ad	GVAX	RCC	Null	Subcutaneous injection or intramuscular injection	IV	NCT00258687
Ad	ColoAd1	RCC	Null	Intravenous administration or intratumoral injection	I	NCT02053220
Ad	adenovirus p53 dendritic cell vaccine SC	Progressive or recurrent metastatic cancer	Null	Subcutaneously injection	II	NCT00704938
Ad	DNX-2440	RCC	Null	Intratumoral injection	I	NCT04714983
Ad	Ad-p53	solid tumors	ICIs	Intratumoral injection	II	NCT03544723
Ad	CG0070	BC	Null	Bladder administration	I	NCT00109655
Ad	CG0070	NMIBC	Null	Bladder administration	I/II	NCT01438112
Ad	CG0070	NMIBC	Null	Not applicable	III	NCT06111235
Ad	CG0070	NMIBC	Null	Not applicable	Not applicable	NCT06443944
Ad	Ad-p53	BC	Null	Bladder injections	I	NCT00003167
VV	PANVAC	NMIBC	BCG	Not applicable	II	NCT02015104
Coxsackievirus	CVA21	NMIBC	mitomycin C	Bladder drip	I	NCT02316171
Reovirus	REOLYSIN^®^	MIBC	Gemcitabine and Cisplatin	Intratumoral administration	1b	NCT02723838
Ad	ETBX-071/ETBX-061/ETBX-051	mCRPC	Null	Subcutaneously injection	I	NCT03481816
Ad	Adenovirus/PSA Vaccine	PC	ADT	Subcutaneously injection	II	NCT00583752
Ad	Adenovirus/PSA Vaccine	PC	Null	Subcutaneously injection	II	NCT00583024
Ad	ETBX-011, ETBX-061 and ETBX-051	solid tumors	Null	Subcutaneously injection	I	NCT03384316
Ad	AdNRGM	PC	CB1954	Intravenous administration	I	NCT04374240
Ad	Ad.hIL-12	PC	Null	Prostate injection	I	NCT00110526
Ad	ADV/RSV-tk	PC	Brachytherapy	Prostate injection	I/II	NCT01913106
Ad	AD5-SGE-REIC/Dkk-3	PC	Null	Prostate injection	1/2a	NCT01931046
Ad	M-VM3	PC	Null	Prostate injection	I	NCT02654938
Ad	Ad5-yCD/mutTKSR39rep-hIL12	PC	Null	Prostate injection	I	NCT02555397
Ad	Adenoviral vector delivery of the IL-12 gene	PC	Null	Prostate injection	I	NCT00406939
Ad	M-VM3	PC	Null	Prostate injection	Ib2	NCT02844699
Ad	CV787/CG7870	PC	Null	Intravenous administration	I/II	NCT00116155
Ad	ChAdOx1.5T4-MVA.5T4	PC	Null	Bladder instillation	I	NCT02390063
Ad	Ad5-yCD/mutTKSR39rep-ADP	PC	IRMT	Not applicable	II	NCT00583492
Ad	Ad-sig-hMUC-1/ecdCD40L	PC	Null	Subcutaneously injection	I	NCT02140996
Ad	VTP850	PC	ADT	Not applicable	I/II	NCT05617040
Ad	Ad-REIC/DKK-3	PC	Null	Prostate injection	I	NCT01197209
Ad	ORCA-010	PC	Null	Intratumoral administration	I/IIa	NCT04097002

#### 3.1.2 Encephalomyocarditis virus and renal cell carcinoma

In recent years, a few studies were focused on the treatment of RCC using the EMCV. In 2010, Roos et al. reported that EMCV treatment rapidly reduces clear-cell RCC (CCRCC) growth (34). They reported that hypoxia-inducible factor (HIF) increases the NF-κB-mediated antiapoptotic response in CCRCC and the inactivation of NF-κB weakens the toxicity of EMCV by triggering rapid apoptosis of infected cells, limiting viral replication, and leading to apoptosis of tumor cells. Immunohistochemical analysis of xenograft tumors showed that the necrotic area of tumors treated with EMCV was much larger and more prominent than that in the control group.

#### 3.1.3 Reovirus and renal cell carcinoma

Reoviruses target tumor cells and generally do not cause notable symptoms in humans after infection; therefore, they have been widely used in tumor research. In most tumor cells, RAS is abnormally overexpressed, which promotes tumor growth and creates conditions for the oncolytic effects of the reovirus. Abnormally activated RAS signaling pathways inhibit the normal function of double-stranded RNA-dependent protein kinase (PKR), preventing its ability to inhibit virus replication by phosphorylating eukaryotic translation initiation factor 2α. This allows the viral genome to be freely transcribed and translated into tumor cells without being hindered by host defense mechanisms (51). In contrast, in non-cancerous cells, PKR can effectively recognize reoviral double-stranded RNA, dimerize rapidly, and initiate a defense response, effectively inhibiting viral replication. In addition, activation of the RAS pathway indirectly affects immune responses by promoting the activity of signaling molecules, such as phosphoinositide 3-kinase, mitogen-activated protein kinase, and extracellular signal-regulated kinase, ultimately inhibiting mRNA translation of the pattern recognition receptor RIG-1 and reducing the ability of cells to perceive and resist viral invasion. Reovirus also uses its σ3 protein as a “cloaking device” to hide its double-stranded RNA structure, further evading detection and activation by PKR, enhancing its survival in tumor cells. Lawson et al. reported that the combined treatment with VCN-01 (a proprietary genetically modified oncolytic reovirus) and cyclophosphamide improves the antitumor immune response in patients with mRCC (52). Reovirus has been shown to initiate an innate immune response characterized by the production of pro-inflammatory chemokines, including RANTES, MIP-1-α, MCP-1, KC, IP-10, and MIG, and can produce pro-inflammatory chemokines in a variety of melanoma and prostate cancer cell lines, in addition to their direct oncolytic effects (48, 53, 54). Cyclophosphamide pretreatment effectively depletes immunosuppressive regulatory T cells (Tregs) and myeloid-derived suppressor cells (MDSCs), alleviates the immune inhibition of effector T cells, and enhances the antitumor immune response. Immunohistochemical analysis of xenograft tumors showed that the necrotic area of tumors treated with EMCV was much larger and more prominent than that in the control group.

#### 3.1.4 Adenovirus and renal cell carcinoma

Research on Ads was primarily focused on their oncogenic properties, with limited applications in the treatment of kidney cancer. In 1983, Bernards et al. first constructed a recombinant Ad type 5 (Ad5) in which the E1b region of Ad5 was replaced with that of Ad12. Based on cell infection and analysis, they demonstrated that the recombinant virus effectively replicates in human embryonic kidney and HeLa cells (55).

Subsequently, Guse et al. modified the Ad and constructed Ad5/3-9HIF-Delta24-VEGFR-1-Ig, an oncolytic Ad with a 5/3 chimeric fiber, HRE (9HIF) driving E1 and E1A, and 24 bp deletion in VEGFR-1-Ig in the E3 region. Ad5/3-9HIF-D24-VEGFR-1-Ig exhibited good specificity and oncolytic activity against kidney cancer cells *in vitro* and demonstrated antitumor efficacy in subcutaneous *in vivo* models (56).

Dendritic cells (DCs) are the most powerful full-time antigen presenting cells (APCs) in the body, which can efficiently uptake, process, process and present antigens, immature DCs have strong migration ability, mature DCs can effectively activate naïve T cells, and are at the center of initiating, regulating and maintaining immune responses. Ad-assembled DC vaccines (DCs-CD137L/CAIX) have shown limited therapeutic efficacy in targeting antigens for kidney cancer treatment. Ding et al. combined dendritic cells with an oncolytic Ad to facilitate the entry of dendritic cells into kidney cancer cells, inducing a persistent protective effect against tumors through the generation of memory cell-mediated immune responses (57). OVs enhance the effectiveness of immunotherapy and interleukin-12 (IL12) increases the antitumor activity. Based on combination, the oncolytic Ad OAV-IL-12 was developed to enhance the immunocytotoxic effects of non-replicating Ad-based DC vaccines. Similarly, Fang et al. combined chimeric antigen receptor T (CAR-T) cells with an oncolytic Ad carrying chemokine (C-C motif) ligand 5 (CCL5) and IL12 to create Ad5-ZD55-hCCL5-hIL12 (58). It has been shown that this combination infects and replicates in renal cancer cell lines, demonstrating its ability to suppress tumor proliferation (58). The combination did not reduce but promote the therapeutic effects, prolonging the survival time of mice and increasing survival rates. A variety of adenovirus vectors, including adenovirus-transfected DCs, GVAX, ColoAd1, DNX-2440, and Ad-p53, have been applied in clinical trials for renal cell carcinoma (RCC). Among these trials, some are in Phase I, while the clinical trial for GVAX has progressed to Phase IV (**Table 2**).

OV therapies for kidney cancer have undergone significant developments. Early studies were focused on the genetic modification of viruses, such as VACV and Ad, to enhance their tumor targeting and apoptotic induction capabilities. In recent years, researchers have begun to explore the combined use of OVs with existing drugs such as PD-1 inhibitors and sunitinib, leveraging the synergistic effects between drugs to enhance the treatment efficacy and improve patient survival rates. For example, the combination of VACV JX-594 and PD-1 inhibitors effectively reduced the tumor burden, whereas the combination of Ad with CAR-T cells, chemokines, and cytokines promoted the therapeutic effects. These results provide new strategies for the treatment of kidney cancer. In addition to PD-1 inhibitors, the following modifications and combinations have been explored: CRISPR-Cas9 genome editing: Recent advances in genome editing technologies, such as CRISPR-Cas9, have enabled the precise modification of OV genomes to enhance their safety, targeting, and oncolytic potency (59). This approach has the potential to overcome viral resistance and improve therapeutic outcomes. Combination with chemotherapy: OVs have been combined with chemotherapeutic agents to improve treatment outcomes. For instance, CG8840 adenovirus demonstrated a synergistic antitumor effect when combined with docetaxel in bladder cancer models (22). Similar studies have shown enhanced efficacy when OVs are combined with cisplatin, gemcitabine, or paclitaxel. Nanoparticle-mediated delivery: OVs can be encapsulated within nanoparticles for targeted delivery and protection from immune neutralization. This approach has been used to improve the stability, biodistribution, and efficacy of OVs (49) (**Tables 2**–**4**).

**Table 3:** Combination of oncolytic viruses with antitumor drugs.

Type of cancer	Virus	Virus name	Targeted drug	Reference	Mode of administration	Novel Payload
Renal cell carcinoma	VV	JX-594	ICIs	Park et al. (49)	Intraperitoneal injection	None
Renal cell carcinoma	VV	JX-595	Sunitinib	Park et al. (50)	Intraperitoneal injection	None
Renal cell carcinoma	REO	None	Sunitinib	Lawson et al. (52)	Intraperitoneal injection	None
Renal cell carcinoma	Ad	DCs-CD137L/CAIX	DC	Ding et al. (57)	Peritumorally injected	OAV-IL-12
Renal cell carcinoma	Ad	Ad5-ZD55-hCCL5-hIL12	CAR-T	Fang et al., (58)	Intratumorally administered	CCL5 and IL12
Bladder cancer	CVB	CVA21	Mitomycin C	Annels et al. (60)	Intravesical administration	None
Bladder cancer	CVB	CVA21	Pembrolizumab	Rudin et al. (61)	Intravenous administration	None
Bladder cancer	MRV	T3D-C	Protein 1 (PD-1) inhibitor	Smelser et al. (62)	Intraperitoneal injection	None
Bladder cancer	MRV	RC402 and RP116	NK cell	Lim et al. (63)	Intravesical administration	None

**Table 4:** Oncolytic virus monotherapy.

Type of cancer	Virus name	Virus	Effect	Reference
Renal cell carcinoma	VV-FCU1	VV	Inhibits orthotopic tumor growth	Fend et al. (64)
Renal cell carcinoma	EMCV	EMCV	Causes inactivation of NF-κB	Roos et al. (34)
Renal cell carcinoma	MV-GFP	MV	Antitumor effect	Miest et al. (65)
Renal cell carcinoma	Ad5	Ad	Replicate in tumors	Bernards et al. (55)
Renal cell carcinoma	Ad5/3-9HIF-Delta24-VEGFR-1-Ig	Ad	Antitumor effect	Guse et al. (56)
Renal cell carcinoma	OAV-IL-12	Ad	Enables dendritic cells to enter cancer cells	Ding et al. (57)
Renal cell carcinoma	Ad5-ZD55-hCCL5-hIL12	Ad	Inhibits tumor expansion	Fang et al., (66)
Bladder cancer	CG8840	Ad	Inhibits tumor expansion	Zhang et al. (67)
Bladder cancer	CG0070	Ad	Inhibits tumor expansion	Ramesh et al., (68)
Bladder cancer	Ad.shDCIR	Ad	Improves T cell activity	Hu et al., (69)
Bladder cancer	vAd-VEGFR-3	Ad	Antitumor effect	Hao et al., (70)
Bladder cancer	VACV	OC	Antitumor effect	Potts et al. (71)
Bladder cancer	rVV-TK-53	OC	Induce P53 expression	Fodor et al. (72)
Bladder cancer	CVA21	CVB	Inhibits tumor expansion	Annels et al. (60)
Prostate cancer	Ad5-IL-12	Ad	Inhibits tumor expansion	Nyati et al. (73)
Prostate cancer	AdKi67-C3	Ad	Inhibits tumor expansion	Fang et al., (74)

### 3.2 Application in bladder cancer treatment

**Figure 3:**
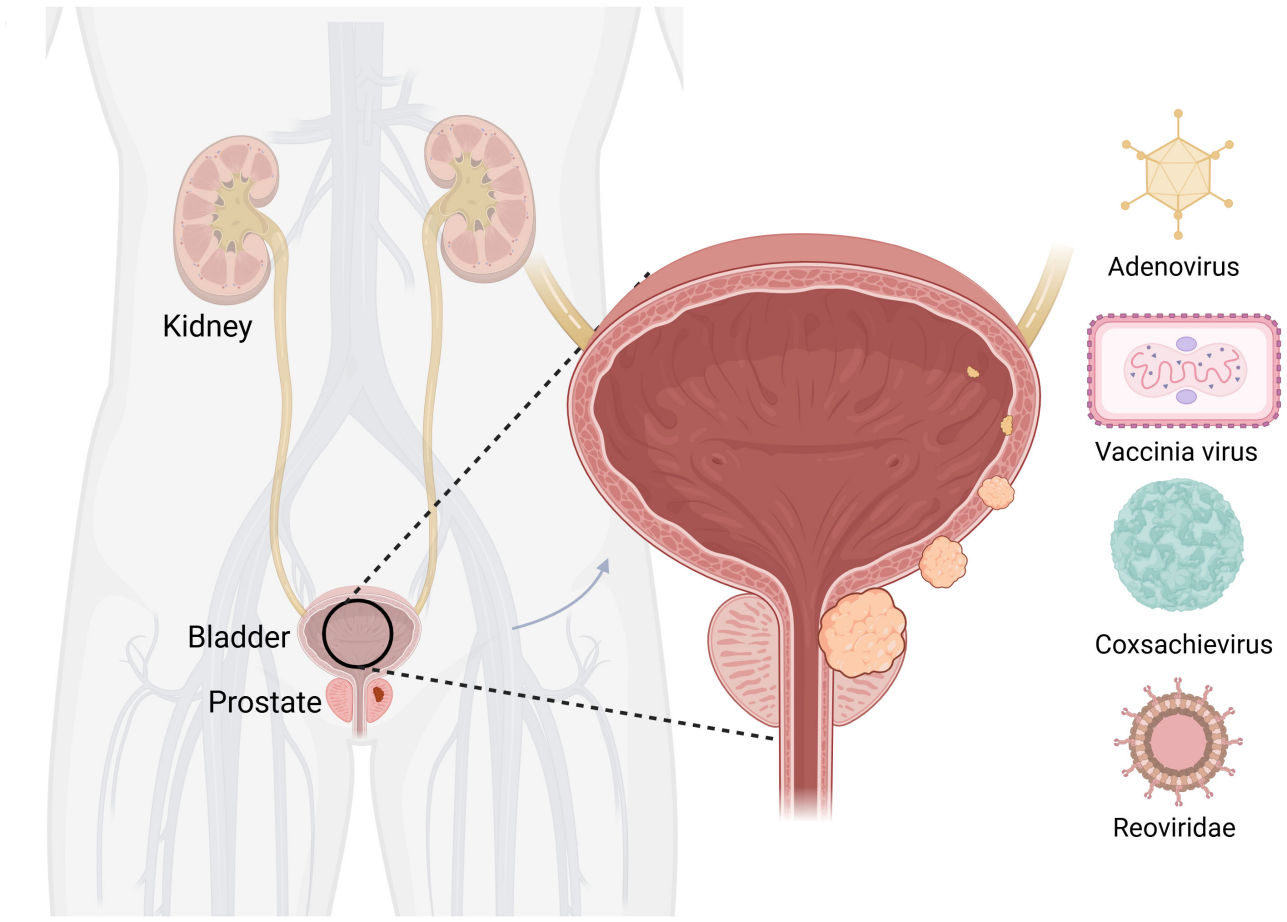
Oncolytic viruses are common in bladder cancer. Created with Biorender.com

Worldwide, BC ranks 10th in the incidence of malignancy. Its main causes are smoking and long-term exposure to industrial chemicals (75). The main treatment methods for non-muscle-invasive BC are surgical treatment and intravesical perfusion, which can be divided into intravesical chemotherapy and intravesical immunotherapy (76). Neoadjuvant chemotherapy combined with radical cystectomy and pelvic lymph node dissection is the mainstay (67). Although various treatment options are available for BC, there is a lack of effective treatments for incurable resectable and metastatic BC. The emergence of OVs provides new ideas for the treatment of BC (**Figure 3**).

#### 3.2.1 Adenovirus and bladder cancer

The treatment efficacy of BC has been improved by partially modifying the genes of Ads or combining them with other treatment modalities. A replication-competent attenuated Ad variant, CG8840, was created by linking a DNA segment upstream of genes expressing urinary tract proteins to a promoterless firefly luciferase reporter gene. The replication capacity of the Ad variant in bladder transitional cell carcinoma (TCC) cells was assessed using the virus yield and 3-(4,5-dimethylthiazol-2-yl)-2,5-diphenyltetrazolium bromide (MTT) assay (68). Compared with non-bladder cells, CG8840 efficiently replicated and eliminated bladder TCC cells with high specificity. In xenograft models of human BC, both the intratumoral and intravenous administration of CG8840 significantly suppressed the tumor growth. When CG8840 was used in combination with docetaxel, a synergistic antitumor effect was observed (68). A marketed oncolytic Ad, CG0070, is used to treat BC (77). CG0070 is based on a modified Ad5 backbone and incorporates a tumor-specific promoter and granulocyte-macrophage colony-stimulating factor (GM-CSF) transgene. It operates through two main mechanisms: 1) it replicates within tumor cells, leading to tumor cell lysis and immunogenic cell death; and 2) the rupture of cancer cells releases tumor-derived antigens and GM-CSF, stimulating systemic antitumor immune responses involving host leukocytes. For the treatment of non-muscle-invasive bladder cancer (NMIBC) that does not respond to interleukin therapy, a Phase II clinical trial of CG0070 in combination with pembrolizumab has been completed. The results of the trial showed that no patients developed muscle-invasive bladder cancer (MIBC) or metastatic bladder cancer, and there were no unintended immune-related adverse effects (78). Both *in vitro* and *in vivo* studies demonstrated that CG0070 possesses selective replication, cytotoxicity, GM-CSF production, and antitumor efficacy in various BC models (77).

In addition to modifications, Ads can be combined with traditional treatment modalities, such as chemotherapy, radiotherapy, and platinum-based drugs, to enhance their effectiveness. CG0070 has been used in clinical trials for BC and NMIBC, including Phase I, Phase I/II, and Phase III (**Table 2**).

#### 3.2.2 Vaccinia virus and bladder cancer

In recent years, there has been limited research on the application of VACV in the treatment of BC. In 2001, Gomella et al. delivered live virus directly to the human bladder for the first time, demonstrating that VACV can be safely administered to the bladder by recruiting lymphocytes and inducing a rapid local inflammatory response (71). To enhance the tumor-specific recognition and cell killing ability of VACV, Potts et al. mutated the F4L and J2R sites, which encodes a viral homolog of the ribonucleotide reductase small subunit (RRM2) involved in cell cycle regulation, to produce a novel oncolytic VACV (72). The tumor selectivity and cell-killing ability of VACV were validated by *in situ* inoculation of human BC cells into rat bladders. Similarly, in 2005, Fodor et al. used a recombinant VACV expressing human p53 to detect the virus’s oncolytic effects its ability to induce p53 transgene-mediated death by assessing the tumor incidence, survival rate, and transgene expression in cultured mouse BC MB-49 cells and cells grown *in situ* in genetically modified mice (79).

#### 3.2.3 Coxsackievirus and bladder cancer

The application of coxsackieviruses in BC treatment typically involves assisting Ads in effectively infecting BC cells (80). Coxsackievirus and Ad receptors (CAR) are considered to be the primary receptors for Ads and are commonly used as gene delivery vectors. The most common coxsackievirus subtype in BC is A21. Coxsackievirus A21 (CVA21) is a novel intercellular adhesion molecule-1 (ICAM-1)-targeted immunotherapeutic virus. Annels et al. investigated the cytotoxicity induced by CVA21 in a series of human BC cell lines, revealing a sensitivity closely associated with the expression of the viral receptor ICAM-1, and studied the ability of CVA21 to induce immunogenic cell death (60). The following year, they completed a phase I trial of CVA21 oncolytic therapy for non-muscle-invasive BC (61). The results showed that, when used alone or in combination with mitomycin C, coxsackievirus led to interferon(IFN) induction, including immune checkpoint inhibitory genes (PD-L1 and LAG3) and Th1-related chemokines, as well as induced the innate activator RIG-I, genes associated with the Th1-mediated immune response, and caused significant inflammatory changes in Non-muscle-invasive bladder cancer (NMIBC) tissue biopsies (61). In 2023, Charles et al. published a study on the safety of the intravenous injection of coxsackievirus A21 (V937) alone or in combination with pembrolizumab in patients with late-stage cancer. They showed that intravenous injection of V937+pembrolizumab is safe; however, in non-small cell lung cancer and BC, its efficacy was not superior to that of previous monotherapy with pembrolizumab, although V937 could be detected in tumor tissue (81). Currently, CVA21 in combination with Mitomycin C has been used in a Phase I clinical trial for NMIBC (**Table 2**).

#### 3.2.4 Reovirus and bladder cancer

The earliest discovery of the ability of bluetongue virus in Reoviridae to produce large amounts of interferon was made by stimulating animals and cell cultures (including human leukocytes) and continuous cell lines (82). In 2003, Kilani et al. first reported preclinical studies of coxsackievirus-mediated oncolysis in BC (83). Hanel et al. were the first to use coxsackievirus *in situ* in a bladder tumor model for the treatment of superficial BC, studying the ability of the virus to kill BC cells *in vitro* and inhibit tumor growth *in vivo* (62). Compared with the complications of the Bacillus Calmette-Guerin Vaccine (BCG vaccine), coxsackieviruses have fewer side effects; the tumor-free survival rate of animals treated with coxsackievirus is significantly higher than that of animals receiving immunotherapy or saline treatment (62). Smelser et al. concluded that the single intravesical administration of coxsackievirus, PD-1 inhibitor, or a combination injection resulted in a higher survival rate in mice with *in situ* bladder tumors compared with the control group (63). Similarly, Lim et al. evaluated the effect of combined treatment with natural killer (NK) cells and coxsackievirus on BC cells using an *in vitro* assay and reported the effective cytotoxicity in metastatic tumor cells (84). Coxsackievirus research started with its ability to produce interferons similar to BC. Subsequently, it was used for the killing of BC. Currently, research leans towards combining it with immunotherapy and testing the effectiveness of this combination.

Ads, VACVs, coxsackieviruses, and reoviruses have been extensively studied and used in BC treatment because of their unique biological properties. These viruses can be engineered to enhance their selectivity for recognizing and killing BC cells, thereby improving the efficacy of BC treatments. OVs yield better treatment outcomes and quality of life for patients with BC. Currently, the clinical application of oncolytic viruses for the treatment of bladder cancer remains limited, thus further research and development are needed to expand their scope of application (**Tables 2**–**4**).

### 3.3 Application in prostate cancer treatment

**Figure 4:**
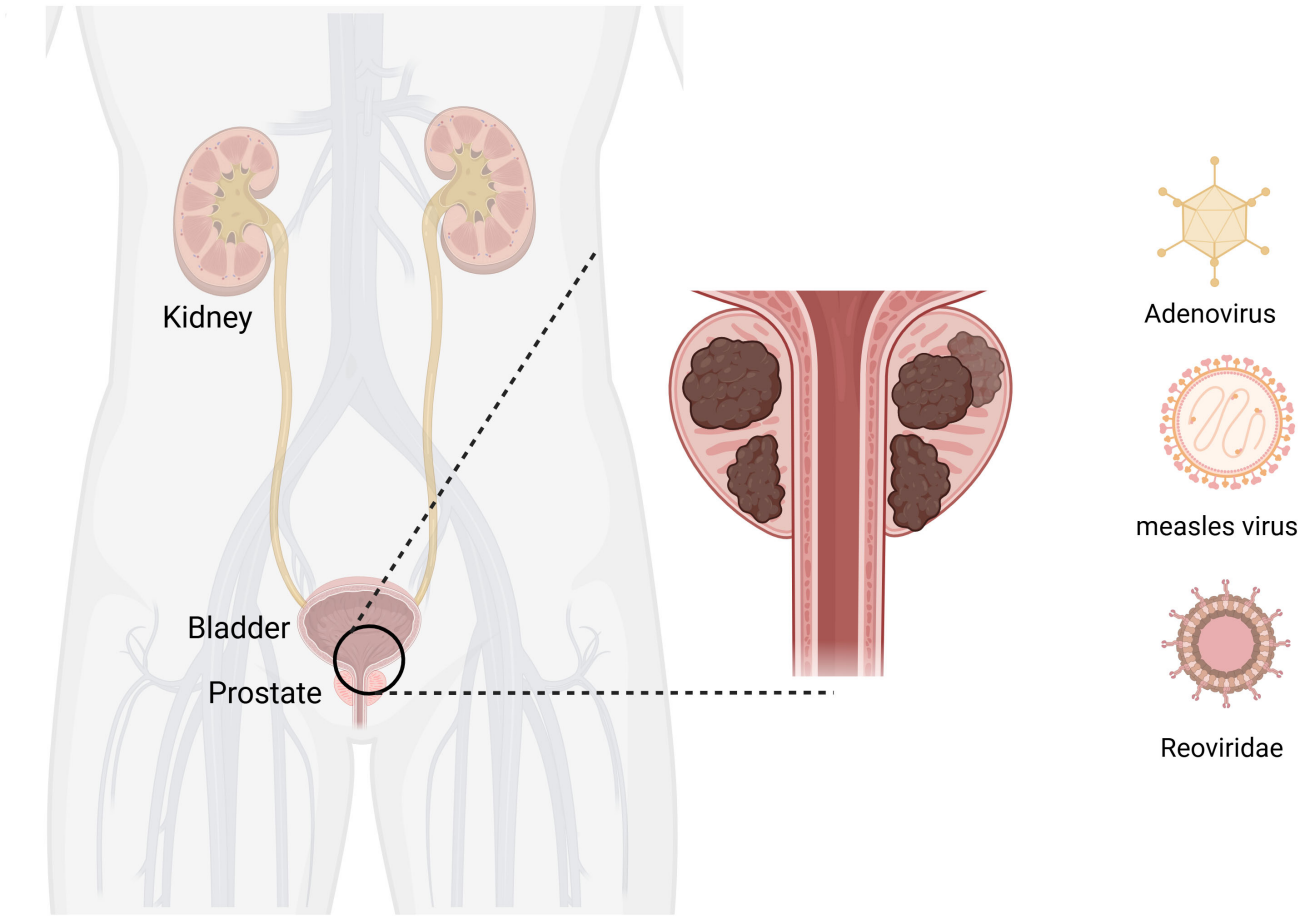
Oncolytic viruses are common in prostate cancer. Created with Biorender.com

PC is the most common malignant tumor of the genitourinary system in men. The incidence of PC in China has significantly increased in recent years (75). Currently, the most recent view is that PC is mainly caused by genetic (85) and sex hormone disorders (86). The primary treatments for organ-limited and locally advanced PC include radical prostatectomy and radiotherapy. Radical resection and radiotherapy are the main treatment methods for PC recurrence after curative therapy (87). Metastatic PC is mainly mediated by androgen deprivation (88). The development of OVs has provided new solutions for the treatment of PC (**Figure 4**).

#### 3.3.1 Adenovirus and prostate cancer

The use of Ads for oncolytic therapy of PC has led to some success. Ads used in the clinical treatment of PC include CV706 (89), CG7870 (90), Ad5-CD/TKrep (FGR (91), and Ad5-yCD/mutTKSR39rep-ADP (73). In addition, many drugs that use oncolytic Ads as a vector for treating PC are still in clinical research. For example, Nyati et al. used an Ad as a vector to deliver suicide and IL12 genes to tumor tissues (92). This trial entered phase I clinical trials and demonstrated a good tolerability when the replication-competent Ad5-IL-12 (Ad5-yCD/mutTKSR39rep-hIL-12) was locally administered to prostate tumors (92). Autio et al. utilized AdC68 vectors to express three selected PC-specific antigens: prostate-specific antigen (PSA), prostate-specific membrane antigen (PSMA), and prostate stem cell antigen (PSCA), along with plasmid DNA (PF-06755990), monoclonal antibodies targeting cytotoxic T-lymphocyte-associated antigen 4 (CTLA-4), pembrolizumab (PF-06753388). This drug has entered phase I trials (NCT02616185). Overall, PF-06753512 a vaccine-based immunotherapy regimen (VBIR) has been declared safe similar to other immune checkpoint inhibitor combination trials; it stimulates antigen-specific immune responses in all cohorts and produces moderate antitumor activity in patients with B-cell receptor (BCR), without the use of ADT (74).

Although many clinical trials have been initiated, the use of Ad as a vector for the treatment of PC is still being investigated. Fang et al. aimed to enhance the efficacy of CAR-T cells against solid tumors. They constructed a novel recombinant oncolytic Ad controlled by the Ki67 promoter, carrying CCL5, IL12, and IFN-γ genes (named AdKi67-C3), which significantly promoted the proliferation and persistence of CAR-T cells *in vitro* and *in vivo* and established long-term antitumor immune responses (93). Gavrikova et al. overcame the shortcomings of control elements and poor infectivity using fiber modification and an androgen-independent promoter (cyclooxygenase-2, COX-2). The results of both *in vitro* and *in vivo* studies showed potent antitumor effects (94). Currently, many Ads are being used in preclinical research. Various adenovirus vectors such as Adenovirus/PSA Vaccine, ETBX-011, ETBX-061, ETBX-051, AdNRGM, Ad-REIC/DKK-3, and ORCA-010 etc. have entered clinical trials for prostate cancer (PC), with trial phases ranging from Phase I to Phase II. Some trials have combined other treatment methods such as ADT, CB1954, and IRMT (**Table 2**). It is evident that there is a relatively large number of adenoviruses currently applied to prostate cancer.

#### 3.3.2 Measles virus and prostate cancer

MV is less commonly used in PC. Recent research was primarily focused on the use of green synthesis-encapsulated attenuated MV to create a novel controllable targeted viral delivery system with ligand-coated surfaces (95). This synthetic virus actively targets cancer cells, protects the virus from antibody clearance, releases OVs via receptor-mediated endocytosis, achieves efficient oncolytic immunotherapy, and enhances targeting (95). Opyrchal et al. studied the effect of actin cytoskeleton regulatory factor inhibition on the oncolytic effect of the MV (96). Msaouel et al. created a MV capable of expressing a human sodium iodide symporter, enabling the virus to induce oncolysis and its use for imaging through iodine-125 (125I) uptake measurements (97).

#### 3.3.3 Enterobacteria and prostate cancer

Enterobacteria can effectively replicate in cells with activated RAS signaling pathways. More importantly, untransformed cells are not sensitive to enterobacteria, indicating the selective infectivity of the virus. Coffey et al. made an exciting discovery: a single intratumoral injection of enterobacteria led to the regression of 65%–80% of tumors in mice (98).

The use of Ads has led to significant progress in the treatment of PC and good potential has been demonstrated in clinical trials. Currently, various Ads are used for the oncolytic therapy of PC including CV706, CG7870, Ad5-CD/TKrep (FGR), and Ad5-yCD/mutTKSR39rep-ADP. These viruses treat PC through different mechanisms such as direct destruction of cancer cells, activation of the immune system, or delivery of anticancer genes. Further research and development of these viruses will lead to more secure, effective, and personalized treatment options for patients with PC (**Tables 2**–**4**).

## 4 Conclusion and challenges

The use of OVs as a potential treatment modality for urological tumors has witnessed a surge in advancement and research. Widely studied OVs include VACV (99), EMCV (100), Ad (101), MV (102), coxsackievirus (103), and reovirus (104), all of which have unique properties and mechanisms of action. The core of research in this area revolves around the genetic modification of these viruses, with the aim of minimizing their adverse effects on healthy cells while maximizing their ability to specifically target and eradicate tumor cells. As a result of these efforts, several OVs have transitioned from laboratory to clinical settings, providing cancer patients novel immunotherapeutic options. For example, the oncolytic Ad CG0070, which is currently in phase II clinical trials, targets bladder tumor cells via a defective retinoblastoma pathway, providing a new solution for patients with BCG-unresponsive non-muscle-invasive BC (105). Recent clinical trials yielded promising results, highlighting the efficacy of OVs in treating urological malignancies (61, 81). This emerging modality represents a ray of hope for cancer patients, providing a potential alternative to traditional treatment methods.

With the advancements in technology and further research, the design of OVs has become increasingly refined. Oncolytic viruses (OVs) are able to trigger cell lysis when they replicate within cancer cells, and as the virus replicates, the number of viruses increases, thus enhancing the destructive power to tumors. At the same time, proteins produced during viral replication also have toxic effects on tumor cells. In addition to directly killing tumor cells, oncolytic viruses can also function through two immune mechanisms: one is to induce a non-specific immune response, and the other is to activate a specific anti-tumor immune response. In addition, oncolytic viruses can significantly increase the sensitivity of tumor cells to chemotherapy and radiotherapy, thereby enhancing the efficacy of these traditional treatments. Precision engineering enables the targeted delivery of viruses to tumor cells, minimizing collateral damage to healthy tissues. The mode of administration of oncolytic viruses is a crucial factor influencing their efficacy and safety in treating genitourinary tumors. Oncolytic viruses can be administered via various routes, including systemic delivery (such as intravenous injection) and loco-regional delivery (such as intratumoral or intravesical injection). Systemic administration enables widespread distribution of the virus throughout the body, which can be beneficial for metastatic tumors. However, it also increases the risk of systemic toxicity. In contrast, loco-regional administration allows for targeted delivery to specific tumor sites, minimizing off-target effects and potentially enhancing the local immune response. For instance, intravesical administration of oncolytic viruses for bladder cancer has shown promising results while minimizing systemic side effects. Therefore, the choice of administration route is guided by the type and stage of the tumor, as well as the patient’s overall health condition. As clinical trials progress, a deeper understanding of the underlying mechanisms and optimal treatment protocols for oncolytic viral therapies will emerge. This knowledge provides a solid foundation for the clinical application of these viruses and their integration into existing treatment paradigms. However, despite the remarkable progress achieved in OV therapy, several challenges remain: 1) Ensuring the safety of the viruses and minimizing potential adverse events remain crucial; 2) Reducing the treatment cost is essential to provide access to a wider patient population; 3) Exploring the synergistic potential of combining OVs with other therapies, such as chemotherapy or immunotherapy, holds promise for enhancing treatment outcomes. In the future, with the continuous advancement of technology and the deepening of clinical trials, the design of oncolytic viruses will be more precise, able to target tumor cells more effectively, and reduce the damage to normal cells. In addition, a better understanding of the mechanism of action of oncolytic virus therapy will help to develop personalized treatment plans to provide the most suitable treatment option for each patient. Although oncolytic virus therapy has shown great potential in the field of urological cancer treatment, it still needs interdisciplinary cooperation, continuous funding and policy support to achieve its wide clinical application. Through these efforts, oncolytic virus therapy is expected to become one of the important means of urinary cancer treatment, bringing new hope and options to patients. Ongoing research, development, and clinical applications are imperative to address these challenges and further advance the field (**Table 2**).

In conclusion, OV therapy is a promising new cancer treatment modality. Although several challenges remain, its prospects are promising considering continuous technological advancements and more detailed clinical investigations.

## Glossary

Ad: adenovirus
ADT: androgen deprivation therapy
BC: bladder cancer
BCR: B-cell receptor
CAR-T: chimeric antigen receptor T
CCRCC: clear cell renal carcinoma
COX-2: cyclooxygenase-2
CTLA-4: cytotoxic T-lymphocyte-associated antigen 4
DCs: dendritic cells
EMCV: encephalomyocarditis virus
GM-CSF: granulocyte-macrophage colony-stimulating factor
IL12: interleukin-12
MDSCs: myeloid-derived suppressor cells
MTT: 3-(4,5-dimethylthiazol-2-yl)-2,5-diphenyltetrazolium bromide
MV: measles virus
NK: natural killer
NMIBC: non-muscle-invasive bladder cancer
OVs: oncolytic viruses
PC: prostate cancer
PD-1: programmed cell death protein 1
PKR: double-stranded RNA-dependent protein kinase
PSA: prostate-specific antigen
PSCA: prostate stem cell antigen
PSMA: prostate-specific membrane antigen
RCC: renal cell carcinoma
RR: ribonucleotide reductase
TCC: transitional cell carcinoma
TK: thymidine kinase
TURBT: transurethral resection of bladder tumor
VACV: vaccinia virus
VBIR: vaccine-based immunotherapy regimen
